# *In Vitro* Activity of Cefepime-Enmetazobactam against Gram-Negative Isolates Collected from U.S. and European Hospitals during 2014–2015

**DOI:** 10.1128/AAC.00514-19

**Published:** 2019-06-24

**Authors:** Ian Morrissey, Sophie Magnet, Stephen Hawser, Stuart Shapiro, Philipp Knechtle

**Affiliations:** a IHMA Europe Sàrl, Monthey, Switzerland; b Allecra Therapeutics SAS, St-Louis, France

**Keywords:** AAI101, ESBL, beta-lactamase inhibitor, cefepime, enmetazobactam, surveillance studies

## Abstract

Enmetazobactam, formerly AAI101, is a novel penicillanic acid sulfone extended-spectrum β-lactamase (ESBL) inhibitor. The combination of enmetazobactam with cefepime has entered clinical trials to assess safety and efficacy in patients with complicated urinary tract infections. Here, the *in vitro* activity of cefepime-enmetazobactam was determined for 1,993 clinical isolates of Enterobacteriaceae and Pseudomonas aeruginosa collected in the United States and Europe during 2014 and 2015. Enmetazobactam at a fixed concentration of 8 μg/ml lowered the cefepime MIC_90_ from 16 to 0.12 μg/ml for Escherichia coli, from >64 to 0.5 μg/ml for Klebsiella pneumoniae, from 16 to 1 μg/ml for Enterobacter cloacae, and from 0.5 to 0.25 μg/ml for Enterobacter aerogenes. Enmetazobactam did not enhance the potency of cefepime against P. aeruginosa. Applying the Clinical and Laboratory Standards Institute susceptible-dose-dependent (SDD) breakpoint of 8 μg/ml to cefepime-enmetazobactam for comparative purposes resulted in cumulative inhibitions of 99.9% for E. coli, 96.4% for K. pneumoniae, 97.0% for E. cloacae, 100% for E. aerogenes, 98.1% for all Enterobacteriaceae assessed, and 82.8% for P. aeruginosa. Comparator susceptibilities for all Enterobacteriaceae were 99.7% for ceftazidime-avibactam, 96.2% for meropenem, 90.7% for ceftolozane-tazobactam, 87% for cefepime (SDD breakpoint), 85.7% for piperacillin-tazobactam, and 81.2% for ceftazidime. For the subset of ESBL-producing K. pneumoniae isolates, the addition of 8 μg/ml enmetazobactam to cefepime lowered the MIC_90_ from >64 to 1 μg/ml, whereas the shift for 8 μg/ml tazobactam was from >64 to 8 μg/ml. Cefepime-enmetazobactam may represent a novel carbapenem-sparing option for empirical treatment of serious Gram-negative infections in settings where ESBL-producing Enterobacteriaceae are expected.

## Abstract (precis)

Enmetazobactam, formerly AAI101, is a novel penicillanic acid sulfone extended-spectrum β-lactamase (ESBL) inhibitor. The combination of enmetazobactam with cefepime has entered clinical trials to assess safety and efficacy in patients with complicated urinary tract infections.

## INTRODUCTION

Third-generation cephalosporin (3GC)-resistant Enterobacteriaceae have been categorized as “critical priority” pathogens (1). Escherichia coli and Klebsiella pneumoniae are among the most frequently isolated pathogens in health care-associated infections across diverse geographies, and the number of deaths attributable to those species rank highest in the United States and Europe (2–5). Novel therapeutic modalities targeting those species are needed urgently.

β-Lactamase enzymes are major contributors of 3GC resistance (6). During the past two decades the CTX-M family of extended-spectrum β-lactamases (ESBLs) has become the dominant mechanism of 3GC-resistance in K. pneumoniae and E. coli (7). The rapid spread of CTX-M-producing Enterobacteriaceae has contributed to an increase in carbapenem consumption, which in turn promotes selection of carbapenem resistance (8–10).

**FIG 1:**
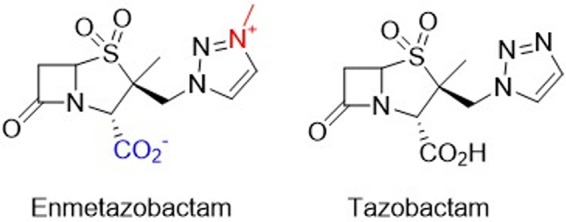
Structures of enmetazobactam and tazobactam. The zwitterionicity of enmetazobactam is highlighted in color.

Enmetazobactam (formerly known as AAI101) is a novel ESBL inhibitor (Fig. 1). It exerts potent inhibitory activity toward CTX-M, TEM, SHV, and other class A β-lactamases through a different mechanism of action than tazobactam (11). Cefepime is a fourth-generation cephalosporin stable to AmpCs and OXA-48 with well-documented efficacy in serious Gram-negative infections (12–14). Against a collection of cefepime-nonsusceptible Enterobacteriaceae, the combination of enmetazobactam with cefepime demonstrated *in vitro* and *in vivo* activity comparable to that of meropenem (15, 16). Cefepime-enmetazobactam is intended as a therapy for infections by ESBL-, AmpC-, and OXA-48-producing strains of Enterobacteriaceae and by Pseudomonas aeruginosa and is expected to provide an empirical treatment option in settings with a high incidence of ESBL-producing Enterobacteriaceae that pursue carbapenem-sparing strategies. In 2018 a multicenter, randomized, double-blind, noninferiority study was initiated comparing cefepime-enmetazobactam with piperacillin-tazobactam in adults with complicated urinary tract infections (cUTI), including acute pyelonephritis (AP) (17).

This surveillance study assessed the *in vitro* activities of cefepime-enmetazobactam and comparator agents against a collection of 1,993 clinical isolates comprised of E. coli, K. pneumoniae, *Enterobacter* spp., and P. aeruginosa. Isolates were collected during 2014 and 2015 in the United States and five European countries. Special emphasis was given to characterization of ESBL-producing isolates of Enterobacteriaceae. In addition, enmetazobactam was compared to tazobactam when combined with cefepime against a subset of ESBL-producing isolates of K. pneumoniae.

## RESULTS AND DISCUSSION

### The data set consisted of 1,993 clinical isolates of Gram-negative pathogens recovered from patients with serious, health care-associated infections.

The species distribution was 35% E. coli, 40% K. pneumoniae, 10% *Enterobacter* spp. (5% E. aerogenes and E. cloacae), and 15% P. aeruginosa. The proportion of K. pneumoniae isolates was inflated relative to its clinical prevalence in order to capture sufficient ESBL-producing isolates, a key target for cefepime-enmetazobactam. Half of the isolates were collected from the United States and half from Europe, with 10% each from Germany, France, Spain, Italy, and the United Kingdom. Genotyping E. coli and K. pneumoniae isolates with a cefepime MIC of ≥1 μg/ml identified 265 strains containing genes encoding ESBLs, Klebsiella pneumoniae carbapenemases (KPCs), metallo-β-lactamases (MBLs), AmpC-β-lactamases (AmpCs), and/or OXA-β-lactamases (OXAs). Among these 265 isolates CTX-Ms were detected in 91.2% of E. coli and 64.9% of K. pneumoniae, followed by 29.8% KPCs, 17.2% SHVs, and 11.3% OXAs in K. pneumoniae (Table 1). More than one β-lactamase was detected in 7.9% of the E. coli isolates and in 23.2% of the K. pneumoniae isolates.

**TABLE 1:** Genotyped β-lactamases and combinations in ESBL-positive isolates of E. coli (*n* = 114) and K. pneumoniae (*n* = 151), excluding non-ESBL SHVs and TEMs

β-Lactamase^*a*^	No. of isolates					
	No additional β-lactamase		Additional CTX-M β-lactamase		Additional SHV β-lactamase	
	E. coli	K. pneumoniae	E. coli	K. pneumoniae	E. coli	K. pneumoniae
CTX-M	96	75	2			4^*c*^
SHV	4	10				
TEM	1		1			
KPC	2	30	1^*b*^	6^*d*^		9^*b*^
VIM		1				1
AmpC	2		4	1	1	
OXA				12		2

a The β-lactamase genes identified included CTX-M-1, -9, -14, -15, -22, -27, -32, -61, -55, and -181; SHV-2, -2A, -7, -12, and -28; TEM-24 and -28; KPC-2 and -3; VIM-1; and the AmpCs CMY-type, ACC-1, DHA-7; and OXA-48 and -232.

b One isolate with an additional AmpC.

c Two isolates with an additional OXA.

d One isolate with an additional OXA.

### Cefepime-enmetazobactam showed potent activity against Gram-negative pathogens.

MIC distributions for cefepime and cefepime-enmetazobactam against all tested pathogens are shown in Table 2. MICs for cefepime-enmetazobactam were determined using a fixed enmetazobactam concentration of 8 μg/ml. For the complete Enterobacteriaceae panel of 1,696 isolates, the addition of enmetazobactam to cefepime lowered the MIC_90_ compared to cefepime alone by seven doubling dilutions from 32 to 0.25 μg/ml. The same MIC_90_ diminution was observed for E. coli isolates, with a shift from 16 to 0.12 μg/ml. The MIC_90_s for K. pneumoniae were reduced by at least eight doubling dilutions from >64 to 0.5 μg/ml. E. cloacae and *E. aerogenes* MIC_90_s were reduced by four and by one doubling dilution, from 16 to 1 μg/ml and from 0.5 to 0.25 μg/ml, respectively. Enmetazobactam did not enhance the potency of cefepime against P. aeruginosa, the MIC_90_ for both cefepime and cefepime-enmetazobactam being 16 μg/ml. Enmetazobactam did not show intrinsic activity against Enterobacteriaceae or P. aeruginosa (data not shown).

**TABLE 2:** Cumulative percentage MIC distribution and ECOFF values of cefepime and cefepime-enmetazobactam against Gram-negative pathogens collected worldwide in the United States and Europe during 2014 and 2015

Species (*n*) and drug	Cumulative % isolates at or below various MICs (μg/ml)^*a*^														ECOFF (μg/ml)
	0.015	0.03	0.06	0.12	0.25	0.5	1	2	4	8	16	32	64	>64	
Enterobacteriaceae															
All (1,696)															
Cefepime	2.7	30.2	62.9	72.9	78.2	81.1	82.5	83.7	85.3	87.0	89.2	**91.5**	93.6	100	
Cefepime-enmetazobactam	2.7	39.6	77.1	87.7	**92.6**	94.8	96.2	96.8	97.3	98.1	98.5	99.0	99.4	100	
															
E. coli (697)															
Cefepime	2.3	27.3	64.3	75.5	80.9	83.2	84.4	85.8	87.8	89.8	**92.3**	94.7	97.1	100	0.12
Cefepime-enmetazobactam	3.6	44.0	86.1	**95.0**	98.9	99.3	99.7	99.9	99.9	99.9	99.9	100			
															
E. coli ESBL genotype^*b*^ (109)															
Cefepime				0.9	0.9	3.7	6.4	13.8	23.9	36.7	52.3	67.0	81.7	**100**	
Cefepime-enmetazobactam	0.9	19.3	69.7	**90.8**	98.2	98.2	99.1	99.1	99.1	99.1	99.1	100			
															
K. pneumoniae (799)															
Cefepime	3.1	35.2	65.1	73.5	77.5	79.5	80.6	80.9	81.2	82.7	85.0	87.6	90.0	**100**	0.12
Cefepime-enmetazobactam	2.3	40.3	76.2	85.0	89.4	**92.4**	93.2	93.7	95.0	96.4	97.2	98.1	99.0	100	
															
K. pneumoniae ESBL genotype^*b*^ (102)															
Cefepime							2.0	3.9	6.9	16.7	30.4	42.2	52.9	**100**	
Cefepime-enmetazobactam		8.8	42.2	66.7	77.5	89.2	**92.2**	93.1	98.0	100					
Cefepime-tazobactam	1.0	8.8	32.4	49.0	60.8	68.6	76.5	85.3	88.2	**92.2**	95.1	96.1	97.1	100	
															
K. pneumoniae KPC genotype^*c*^ (45)															
Cefepime										2.2	11.1	28.9	46.7	**100**	
Cefepime-enmetazobactam						4.4	6.7	13.3	24.4	42.2	57.8	71.1	86.7	**100**	
															
E. aerogenes (100)															
Cefepime	5.0	35.0	62.0	69.0	81.0	**91.0**	96.0	97.0	99.0	100					0.12
Cefepime-enmetazobactam	2.0	35.0	64.0	84.0	**94.0**	97.0	99.0	100							
															
E. cloacae (100)															
Cefepime		7.0	37.0	55.0	62.0	69.0	71.0	79.0	86.0	88.0	**90.0**	91.0	91.0	100	0.25
Cefepime-enmetazobactam		7.0	34.0	63.0	74.0	81.0	**92.0**	96.0	96.0	97.0	98.0	98.0	98.0	100	
															
P. aeruginosa (297)															
Cefepime				0.3	1.3	2.7	12.1	45.1	64.3	79.5	**92.3**	95.6	98.3	100	16
Cefepime-enmetazobactam				0.7	1.0	2.0	12.1	44.8	67.3	82.8	**93.6**	96.3	98.3	100	

a MIC_90_ values are in boldface.

b Isolates containing an ESBL gene with or without OXA-48 and/or AmpC genes.

c Isolates containing a KPC gene with or without ESBL, OXA-48, and/or AmpC genes.

The epidemiological cutoff (ECOFF) values for cefepime were determined for each species (18) and are reported in Table 2. Against E. coli and K. pneumoniae, the ECOFF values were 0.12 μg/ml. The ECOFF values for *E. aerogenes* and E. cloacae were 0.12 and 0.25 μg/ml, respectively, and 16 μg/ml for P. aeruginosa.

### Enmetazobactam restored the activity of cefepime against ESBL-producing isolates of *E. coli* and *K. pneumoniae*.

For ESBL-producing isolates of E. coli, enmetazobactam lowered the cefepime MIC_90_ by at least ten doubling dilutions from >64 to 0.12 μg/ml and for ESBL-producing K. pneumoniae by at least seven doubling dilutions from >64 to 1 μg/ml (Table 2). Applying the 2019 Clinical and Laboratory Standards Institute (CLSI) susceptible-dose dependent (SDD) breakpoint for cefepime of 8 μg/ml, enmetazobactam shifted all but one ESBL-producing isolates from the resistant category to the susceptible category, thereby restoring the activity of cefepime toward these species. Cefepime-enmetazobactam had only limited activity against K. pneumoniae isolates containing genes encoding KPC (MIC_90_ of >64 μg/ml) and VIM (MICs of >64 μg/ml) carbapenemases.

### Enmetazobactam is more potent than tazobactam against ESBL-producing isolates of *K. pneumoniae*.

The activities of enmetazobactam and tazobactam, both at fixed concentrations of 8 μg/ml, were compared in combination with cefepime against the subset of ESBL-producing isolates of K. pneumoniae (Fig. 1 and Table 2). Enmetazobactam shifted the MIC_90_ of cefepime from >64 μg/ml to 1 μg/ml, whereas the shift for tazobactam was from >64 μg/ml to 8 μg/ml.

### Activity of cefepime-enmetazobactam versus comparators.

The percentages of susceptible isolates (Table 3) were determined for the β-lactam antibiotics cefepime, ceftazidime, and meropenem; the β-lactam/β-lactamase inhibitor combinations piperacillin-tazobactam, ceftolozane-tazobactam, and ceftazidime-avibactam; the aminoglycoside gentamicin; and the fluoroquinolone ciprofloxacin using 2019 CLSI and EUCAST breakpoints (19, 20). For cefepime-enmetazobactam, cefepime breakpoints ranging from 1 μg/ml (the EUCAST susceptible category) to 8 μg/ml (the CLSI SDD category) were applied for comparative purposes only.

**TABLE 3:** Activities of cefepime-enmetazobactam and comparator agents tested against clinical Gram-negative isolates

Species (*n*), drug, and region	MIC (μg/ml)			% susceptible	
	MIC_50_	MIC_90_	Range	CLSI	EUCAST
Enterobacteriaceae					
All (1,696)					
Cefepime	0.06	32	0.015 to >64	83.7	82.5
Cefepime-enmetazobactam	0.06	0.25	0.015 to >64	NA*^c^*	NA
Piperacillin-tazobactam	2	64	0.12 to >128	85.7	82.0
Meropenem	0.03	0.06	0.008 to >8	96.2	96.4
Ceftolozane-tazobactam	0.25	2	0.06 to >32	90.7	88.5
Ceftazidime	0.25	64	0.03 to >64	81.2	77.7
Ceftazidime-avibactam	0.12	0.5	≤0.015 to >64	99.7	99.7
Gentamicin	0.5	16	0.12 to >32	89.0	88.3
Ciprofloxacin	0.03	>16	0.004 to >16	71.7	71.7
United States (848)					
Cefepime	0.06	4	0.015 to >64	88.6	87.9
Cefepime-enmetazobactam	0.06	0.25	0.015 to >64	NA	NA
Piperacillin-tazobactam	2	32	0.12 to >128	89.5	86.2
Meropenem	0.03	0.03	0.008 to >8	97.8	97.8
Ceftolozane-tazobactam	0.25	1	0.06 to >32	93.8	91.4
Ceftazidime	0.25	32	0.03 to >64	86.1	83.4
Ceftazidime-avibactam	0.12	0.25	≤0.015 to 16	99.9	99.9
Gentamicin	0.5	2	0.12 to >32	90.9	90.3
Ciprofloxacin	0.03	>16	0.004 to >16	75.4	75.4
Europe (848)					
Cefepime	0.06	>64	0.015 to >64	78.9	77.1
Cefepime-enmetazobactam	0.06	0.25	0.015 to >64	NA	NA
Piperacillin-tazobactam	2	>128	0.25 to >128	81.8	77.8
Meropenem	0.03	0.06	0.008 to >8	94.7	95.0
Ceftolozane-tazobactam	0.25	4	0.06 to >32	87.6	85.6
Ceftazidime	0.25	64	0.06 to >64	76.3	71.9
Ceftazidime-avibactam	0.12	0.5	≤0.015 to >64	99.5	99.5
Gentamicin	0.5	32	0.12 to >32	87.1	86.3
Ciprofloxacin	0.03	>16	0.004 to >16	68.0	68.0
E. coli (697)					
Cefepime	0.06	16	0.015 to >64	85.8	84.4
Cefepime-enmetazobactam	0.06	0.12	0.015 to 32	NA	NA
Piperacillin-tazobactam	2	8	≤0.12 to >128	92.4	90.5
Meropenem	0.015	0.03	0.008 to 8	99.6	99.7
Ceftolozane-tazobactam	0.25	0.5	0.06 to >32	98.1	96.8
Ceftazidime	0.25	16	0.06 to >64	86.7	82.2
Ceftazidime-avibactam	0.12	0.25	≤0.015 to 2	100	100
Gentamicin	0.5	32	0.12 to >32	86.2	85.5
Ciprofloxacin	0.015	>16	0.004 to >16	64.1	64.1
E. coli ESBL genotype^*a*^ (109)					
Cefepime	16	>64	0.12 to >64	13.8	6.4
Cefepime-enmetazobactam	0.06	0.12	0.016 to 32	NA	NA
Piperacillin-tazobactam	4	64	0.5 to >128	82.6	75.2
Meropenem	0.03	0.03	0.008 to 8	99.1	99.1
Ceftolozane-tazobactam	0.5	2	0.12 to >32	93.6	88.1
Ceftazidime	16	64	1 to >64	26.6	3.7
Ceftazidime-avibactam	0.12	0.25	≤0.015 to 2	100	100
Gentamicin	1	>32	0.12 to >32	59.6	58.7
Ciprofloxacin	>16	>16	0.008 to >16	9.2	9.2
K. pneumoniae (799)					
Cefepime	0.06	>64	0.015 to >64	80.9	80.6
Cefepime-enmetazobactam	0.06	0.5	0.015 to >64	NA	NA
Piperacillin-tazobactam	4	>128	0.25 to >128	83.1	78.6
Meropenem	0.03	0.12	0.008 to >8	92.7	92.9
Ceftolozane-tazobactam	0.25	8	0.06 to >32	87.5	85.7
Ceftazidime	0.25	>64	0.03 to >64	80.4	78.1
Ceftazidime-avibactam	0.12	0.5	≤0.015 to >64	99.6	99.6
Gentamicin	0.25	8	0.12 to >32	90.0	89.1
Ciprofloxacin	0.03	>16	0.004 to >16	75.2	75.2
K. pneumoniae ESBL genotype^*a*^ (102)					
Cefepime	64	>64	1 to >64	3.9	2.0
Cefepime-enmetazobactam	0.12	1	0.03 to 8	NA	NA
Piperacillin-tazobactam	32	>128	1 to >128	44.1	28.4
Meropenem	0.03	1	0.016 to >8	92.2	91.2
Ceftolozane-tazobactam	2	32	0.12 to >32	52.9	47.1
Ceftazidime	64	>64	0.25 to >64	4.9	2.0
Ceftazidime-avibactam	0.25	1	≤0.015 to 2	100	100
Gentamicin	32	>32	0.25 to >32	41.2	38.2
Ciprofloxacin	>16	>16	0.008 to >16	7.8	7.8
K. pneumoniae KPC genotype^*b*^ (45)					
Cefepime	>64	>64	8 to >64	0.0	0.0
Cefepime-enmetazobactam	16	>64	0.5 to >64	NA	NA
Piperacillin-tazobactam	>128	>128	1 to >128	2.2	2.2
Meropenem	>8	>8	4 to >8	0.0	0.0
Ceftolozane-tazobactam	>32	>32	16 to >32	0.0	0.0
Ceftazidime	>64	>64	32 to >64	0.0	0.0
Ceftazidime-avibactam	1	4	0.03 to >16	97.8	97.8
Gentamicin	2	>32	0.12 to >32	71.1	66.7
Ciprofloxacin	>16	>16	0.5 to >16	0.0	0.0
E. aerogenes (100)					
Cefepime	0.06	0.5	0.015 to 8	97.0	96.0
Cefepime-enmetazobactam	0.06	0.25	0.015 to 2	NA	NA
Piperacillin-tazobactam	2	64	0.25 to 128	77.0	69.0
Meropenem	0.03	0.12	0.015 to 1	100	100
Ceftolozane-tazobactam	0.25	4	0.06 to 32	84.0	76.0
Ceftazidime	0.25	64	0.06 to >64	73.0	65.0
Ceftazidime-avibactam	0.12	0.5	≤0.015 to 4	100	100
Gentamicin	0.5	0.5	0.12 to >32	98.0	98.0
Ciprofloxacin	0.015	0.12	0.004 to >16	91.0	91.0
E. cloacae (100)					
Cefepime	0.12	16	0.03 to >64	79.0	71.0
Cefepime-enmetazobactam	0.12	1	0.03 to >64	NA	NA
Piperacillin-tazobactam	4	128	1 to >128	68.0	63.0
Meropenem	0.03	0.12	0.008 to >8	97.0	98.0
Ceftolozane-tazobactam	0.5	16	0.25 to >32	71.0	65.0
Ceftazidime	0.5	>64	0.12 to >64	58.0	55.0
Ceftazidime-avibactam	0.25	0.5	0.03 to >64	98.0	98.0
Gentamicin	0.25	0.5	0.12 to >32	92.0	92.0
Ciprofloxacin	0.03	2	0.008 to >16	77.0	77.0
					
P. aeruginosa (297)					
Cefepime	4	16	0.12 to >64	79.5	79.5
Cefepime-enmetazobactam	4	16	0.12 to >64	NA	NA
Piperacillin-tazobactam	8	128	0.12 to >128	75.4	75.4
Meropenem	0.5	>8	0.015 to >8	76.4	76.4
Ceftolozane-tazobactam	0.5	4	0.25 to >32	92.6	92.6
Ceftazidime	4	64	0.25 to >64	78.5	78.5
Ceftazidime-avibactam	2	8	0.06 to >64	95.0	95.0
Gentamicin	2	32	0.12 to >32	84.5	84.5
Ciprofloxacin	0.25	16	0.004 to >16	68.0	68.0
United States (149)					
Cefepime	4	16	0.5 to >64	82.6	82.6
Cefepime-enmetazobactam	4	16	0.12 to >64	NA	NA
Piperacillin-tazobactam	4	64	0.12 to >128	81.2	81.2
Meropenem	0.5	8	0.015 to >8	75.2	75.2
Ceftolozane-tazobactam	0.5	2	0.25 to >32	98.0	98.0
Ceftazidime	4	16	0.5 to 64	87.2	87.2
Ceftazidime-avibactam	2	4	0.25 to >64	98.7	98.7
Gentamicin	2	8	0.12 to >32	89.3	89.3
Ciprofloxacin	0.12	8	0.03 to >16	73.2	73.2
Europe (148)					
Cefepime	4	32	0.12 to >64	76.4	76.4
Cefepime-enmetazobactam	4	32	0.12 to >64	NA	NA
Piperacillin-tazobactam	8	>128	0.25 to >128	69.9	69.9
Meropenem	0.5	>8	0.03 to >8	77.7	77.7
Ceftolozane-tazobactam	0.5	8	0.25 to >32	87.2	87.2
Ceftazidime	4	64	0.25 to >64	69.6	69.6
Ceftazidime-avibactam	2	8	0.06 to >64	91.2	91.2
Gentamicin	2	>32	0.12 to >32	79.6	79.6
Ciprofloxacin	0.25	>16	0.004 to >16	62.8	62.8

a Isolates containing genes encoding an ESBL with or without OXA-48 or AmpC β-lactamases.

b Isolates containing genes encoding a KPC with or without an ESBL, OXA-48, and/or AmpC β-lactamases.

c NA, not applicable.

For the combined Enterobacteriaceae, >90% of isolates were susceptible to meropenem, ceftolozane-tazobactam, and ceftazidime-avibactam according to CLSI criteria. For cefepime, piperacillin-tazobactam, ceftazidime and gentamicin, the susceptibility of isolates ranged from 80 to 90% but was below 80% for ciprofloxacin. Applying cefepime breakpoints of 1 to 8 μg/ml to cefepime-enmetazobactam resulted in cumulative inhibitions of 96.2 to 98.1%, respectively. For each agent tested, the percentage of susceptible Enterobacteriaceae isolates was higher in the United States than in Europe. Applying a breakpoint of 8 μg/ml to cefepime-enmetazobactam resulted in the following country-adjusted, cumulative inhibitions: 100% for France and the United Kingdom, 99.4% for Spain, 98.8% for the United States and Germany, and 88.2% for Italy.

For E. coli, >90% of isolates were in the CLSI susceptible category for piperacillin-tazobactam, meropenem, ceftolozane-tazobactam, and ceftazidime-avibactam. Applying a breakpoint of 1 μg/ml to cefepime-enmetazobactam inhibited 99.7% of all E. coli isolates. For K. pneumoniae, meropenem and ceftazidime-avibactam had >90% of isolates in the CLSI susceptible category. Applying breakpoints of 1 to 8 μg/ml to cefepime-enmetazobactam resulted in cumulative inhibitions of 93.2 to 96.4%, respectively, for all K. pneumoniae isolates. At their CLSI breakpoints, >90% of *E. aerogenes* isolates were susceptible to cefepime, meropenem, ceftazidime-avibactam, gentamicin, and ciprofloxacin, whereas >90% of E. cloacae isolates were susceptible to meropenem, ceftazidime-avibactam, and gentamicin. Susceptibility of E. cloacae to ceftolozane-tazobactam was 71%. Applying breakpoints of 1 to 8 μg/ml to cefepime-enmetazobactam resulted in cumulative inhibitions of 99.0 to 100% for *E. aerogenes* and 92.0 to 97.0% for E. cloacae isolates.

Against the subset of E. coli with an ESBL genotype, only meropenem, ceftolozane-tazobactam, and ceftazidime-avibactam had >90% of isolates in the CLSI susceptible category; for K. pneumoniae with an ESBL genotype, this was the case for meropenem and ceftazidime-avibactam only. Between 50 and 85% susceptible isolates were observed for piperacillin-tazobactam and gentamicin for E. coli, and for ceftolozane-tazobactam for K. pneumoniae. The remaining comparators had less than 45% susceptible isolates by CLSI criteria for E. coli and K. pneumoniae with an ESBL genotype. Applying breakpoints of 1 to 8 μg/ml for cefepime-enmetazobactam resulted in cumulative inhibitions of 99.1% for E. coli and 92.2 to 100% for K. pneumoniae with an ESBL genotype, respectively. The combination of cefepime with tazobactam resulted in cumulative inhibitions of 76.5 to 92.2%, respectively, for ESBL genotype K. pneumoniae.

Against the subset of K. pneumoniae isolates with a KPC genotype, only ceftazidime-avibactam had >90% of isolates in the susceptible category. For gentamicin 71.1% of these isolates were in the CLSI susceptible category and between 0 and 5% for the remaining comparators. Applying breakpoints of 1 to 8 μg/ml for cefepime-enmetazobactam to K. pneumoniae isolates with a KPC genotype resulted in cumulative inhibitions of 6.7 to 42.2%, respectively.

For P. aeruginosa ceftolozane-tazobactam and ceftazidime-avibactam each had >90% of isolates in the CLSI susceptible category, and between 65 and 85% for the remaining comparators. Applying the cefepime breakpoint of 8 μg/ml rendered 82.8% of isolates susceptible to cefepime-enmetazobactam.

### Resistance to 3GCs leaves clinicians with limited empirical treatment options.

Carbapenems are recommended for infections caused by ESBL-producing Enterobacteriaceae (21), which has contributed to the growing carbapenem consumption in high-income countries during the past 2 decades (9). The emergence and spread of carbapenem-resistant pathogens was predictable (8, 22), and carbapenem-resistant infections have become a serious public health threat with ensuing morbidity and mortality (23, 24). Sparing carbapenem usage is advised as part of antimicrobial stewardship programs (10). Piperacillin-tazobactam is a carbapenem-sparing option for infections caused by ESBL-producing E. coli and K. pneumoniae (25, 26). However, the outcomes from the recent MERINO study do not support piperacillin-tazobactam as an alternative to meropenem in patients with bloodstream infections caused by ceftriaxone-resistant E. coli or K. pneumoniae (27).

The present study found that enmetazobactam restored the activity of cefepime, a 4th-generation cephalosporin, against recent United States and European clinical isolates of Enterobacteriaceae expressing diverse ESBLs. Applying the CLSI breakpoint for cefepime to cefepime-enmetazobactam revealed that this novel β-lactam/β-lactamase inhibitor combination outperformed piperacillin-tazobactam and was as potent as meropenem toward the complete Enterobacteriaceae panel and toward the subset of ESBL-producing E. coli and K. pneumoniae isolates, though it showed limited activity against KPC-producing Enterobacteriaceae. The addition of enmetazobactam also enhanced substantially the *in vitro* efficacy of cefepime against E. cloacae, with a much-improved MIC_90_ compared to either piperacillin-tazobactam or ceftolozane-tazobactam and an MIC_90_ comparable to that of ceftazidime-avibactam.

### Conclusion.

The results of this study suggest that cefepime-enmetazobactam may prove to be a valuable carbapenem-sparing option for empirical treatment of serious Gram-negative infections in settings with an elevated prevalence of ESBL-producing Enterobacteriaceae. The intrinsic activity of cefepime against AmpCs and OXA-48 (12, 13) implies that cefepime-enmetazobactam also will be useful for treating infections caused by Enterobacteriaceae expressing these resistance mechanisms in conjunction with an ESBL.

## MATERIALS AND METHODS

Bacteria were isolated from hospitalized patients with cUTI or AP, pneumonia, and intraabdominal infections. Pathogen collection and analysis were performed by IHMA Europe Sàrl (Monthey, Switzerland). The pathogen breakdowns by year 2014/2015 were 48.4%/51.6% for E. coli, 41.4%/58.6% for K. pneumoniae, 20.0%/80% for *E. aerogenes*, 23%/77% for E. cloacae, and 13.5%/86.5% for P. aeruginosa. Only one isolate per patient was included.

Matrix-assisted laser desorption ionization-time of flight mass spectrometry was used to confirm the identity of the organisms (Bruker Daltonics, Bremen, Germany). MICs were determined by broth microdilution according to CLSI guidelines using frozen antimicrobial panels (28). The percentage of isolates susceptible to comparator antibiotics was determined according to 2019 CLSI and EUCAST breakpoints (19, 20). Cefepime-enmetazobactam breakpoints have not yet been assigned. For purposes of comparison CLSI or EUCAST breakpoints for cefepime alone were applied to cefepime-enmetazobactam (see Results section). Quality control tests were performed with E. coli ATCC 25922, E. coli ATCC 35218, K. pneumoniae ATCC 700603, and P. aeruginosa ATCC 27853 each day of testing in compliance with CLSI guidelines (19). Cefepime-enmetazobactam MICs were determined using enmetazobactam at a fixed concentration of 8 μg/ml; likewise, cefepime-tazobactam MICs were determined using tazobactam at a fixed concentration of 8 μg/ml. Quality control ranges of cefepime-enmetazobactam have been approved by the CLSI for the aforementioned quality control strains (29). ECOFF values were determined as described previously (18) using the ECOFFinder_XL_2010_v2.0 file (http://www.eucast.org/mic_distributions_and_ecoffs/) for Microsoft Excel v1812, reporting the ECOFF 99% rounded up to the next MIC.

E. coli and K. pneumoniae isolates with a cefepime MIC of ≥1 μg/ml were genotyped by multiplex PCR for genes encoding class A ESBLs (CTX-M, SHV, and TEM) and KPCs, MBLs (IMP, VIM, NDM, and SPM), AmpCs (ACC, CMY, DHA, FOX, and ACT), and class D (OXA-48-like β-lactamases), followed by sequencing using methods described previously (30). E. coli or K. pneumoniae isolates were classified as having an “ESBL genotype” if an isolate contained a gene encoding an ESBL according to the Bacterial Antimicrobial Resistance Reference Gene Database (31), irrespective of the presence of an AmpC and/or the OXA-48 gene sequence (32). Isolates were classified as having a “KPC genotype” if an isolate contained a gene encoding a KPC irrespective of the presence of an ESBL, AmpC and/or OXA-48 gene sequence.
